# Pathological Evaluation of Rectal Cancer Specimens Using Micro-Computed Tomography

**DOI:** 10.3390/diagnostics12040984

**Published:** 2022-04-14

**Authors:** Masao Yoshida, Emine Cesmecioglu, Canan Firat, Hirotsugu Sakamoto, Alexei Teplov, Noboru Kawata, Peter Ntiamoah, Takashi Ohnishi, Kareem Ibrahim, Efsevia Vakiani, Julio Garcia-Aguilar, Meera Hameed, Jinru Shia, Yukako Yagi

**Affiliations:** 1 Department of Pathology, Memorial Sloan Kettering Cancer Center, New York, NY 10065, USA; eminecesmecioglu@gmail.com (E.C.); firatc@mskcc.org (C.F.); teplov@mskcc.org (A.T.); ntiamoap@mskcc.org (P.N.); ohnishit@mskcc.org (T.O.); ibrahik1@mskcc.org (K.I.); vakianie@mskcc.org (E.V.); hameedm@mskcc.org (M.H.); shiaj@mskcc.org (J.S.); yagiy@mskcc.org (Y.Y.); 2 Division of Endoscopy, Shizuoka Cancer Center, Shizuoka 411-8777, Japan; n.kawata@scchr.jp; 3 Department of Pathology, Marmara University Research and Education Hospital, Istanbul 34899, Turkey; 4 Department of Medicine, Division of Gastroenterology, Jichi Medical University, Tochigi 329-0498, Japan; 94036hs@jichi.ac.jp; 5 Department of Surgery, Memorial Sloan Kettering Cancer Center, New York, NY 10065, USA; garciaaj@mskcc.org

**Keywords:** rectal cancer, micro-CT, whole-block imaging, three-dimensional images

## Abstract

Whole-block imaging (WBI) using micro-computed tomography (micro-CT) allows the nondestructive reconstruction of a three-dimensional view of tissues, implying that WBI may be used for accurate pathological evaluation of patients with rectal cancer. HOWEVER, the clinical impact of this approach is unclear. We aimed to clarify the efficacy of WBI in the whole-mount specimens of locally advanced rectal cancer. A total of 237 whole-mount formalin-fixed paraffin-embedded blocks from 13 patients with rectal cancer who underwent surgical treatment were enrolled and scanned with micro-CT to generate three-dimensional images. WBI was evaluated following the conventional pathological review of the corresponding whole-slide imaging (WSI). WBI identified all tumor sites detected using WSI. Furthermore, WBI revealed one additional tumor site, which was not detected using WSI. Tumor resection margin was significantly closer to the soft-tissue edge when measured using WBI (7.7 mm vs. 6.6 mm, *p* < 0.01). Seventy-six percent of tumor deposits on WSI were changed according to the evidence of tumor interaction with the surrounding tissues confirmed using WBI. Furthermore, WBI revealed 25 additional lymph nodes, six of which were metastatic. The combination of conventional hematoxylin and eosin-stained imaging and WBI may contribute to an accurate pathological assessment.

## 1. Introduction

Colorectal cancer is the third most commonly diagnosed cancer among men and women, with an estimated 1.9 million new cases, and the second leading cause of cancer-related deaths, with an estimated 0.9 million deaths worldwide in 2020 [1]. Rectal cancer accounts for nearly 30% of colorectal cancer cases [2,3], and 40% of the patients with rectal cancer are diagnosed with locally advanced disease [4,5]. Although the 5 year overall survival is slightly higher in patients with rectal cancers than in those with colon cancers [4], the treatment of rectal cancer is more challenging because it affects the anal sphincter, bladder, and sexual function, and a balanced therapeutic approach to preserve patient quality of life is required [6].

With the development of precision medicine in rectal cancer patients, various prognostic factors were investigated, and scoring systems were proposed [7,8,9,10,11,12]. A prognosis prediction model after rectal surgery includes several pathological factors and provides important information when considering an appropriate adjuvant treatment and surveillance intervals. Therefore, an accurate pathological diagnosis of rectal surgery is required since it has important clinical implications on treatment decisions. However, most patients with locally advanced cancer are treated before surgical resection with neoadjuvant combined modality therapy, which makes the histological assessment more complicated [13,14]. The effectiveness of pathological evaluation of the whole-mount specimens in rectal cancer has been reported previously, which enables grasping a general overview from the superficial mucosa to mesorectum [15,16].

Micro-focus X-ray computed tomography (micro-CT) provides a nondestructive reconstruction of high-resolution digital three-dimensional (3D) serial images up to 1 mm from the entire formalin-fixed paraffin-embedded (FFPE) tissue blocks [17,18]. Although micro-CT itself was initially developed for industrial and material science, recent studies have shown the efficacy of histological evaluation in breast, lung, and thyroid cancer using whole-block imaging (WBI) [19,20,21,22]. Because WBI enables pathologists to review entire FFPE blocks in 3D, it may reduce the workload such as a recut for deeper layer evaluation. Simultaneously, whole-slide imaging (WSI) using conventional glass slides provides information on one-side surface of the FFPE block. Therefore, we hypothesized that the combination of WSI and WBI could provide mutually complementary pathological information. The aim of this study was to clarify the efficacy of the additional review of WBI in the whole-mount specimens of locally advanced rectal cancer.

## 2. Materials and Methods

### 2.1. Patients and Materials

A total of 13 patients with rectal cancer who underwent surgical treatment at our institution between March 2018 and January 2020 were randomly chosen from the institutional database. Patient data were retrieved from electronic medical records.

The specimens obtained were fixed in 10% formalin for 24 h at room temperature, sectioned serially, and then embedded in paraffin. Whole-mount slides were prepared as previously described [15,16]. Horizontal sections of 5 mm thickness of the entire specimen at the level of the tumor, including the perirectal soft tissue and distal margin, were cut and stained with hematoxylin and eosin (H&E). Subsequently, a micro-CT scan of FFPE blocks was taken. A total of 237 FFPE blocks and the corresponding 237 H&E slides of the whole-mount specimen were collected.

### 2.2. Whole-Slide Imaging Analysis

For pathological review for WSI, whole-mount H&E glass slides were digitally scanned to create virtual slides using a NanoZoomer S60 (Hamamatsu Photonics, Hamamatsu, Japan) at 20× magnification (0.46 µm/pixel) [23,24]. WSI revealed the following findings: depth of tumor invasion, tumor deposit (TD), circumferential resection margin (CRM), benign lymph nodes (BLN), metastatic lymph nodes (MLN), lymphovascular invasion (LVI), and perineural invasion (PNI). The diagnoses of TD, LVI, and PNI were made according to the American Joint Committee on Cancer (AJCC) staging manual [25]. CRM was measured as the distance from the deepest part of the tumor to the inked soft-tissue edge [9].

### 2.3. Whole-Block Imaging Analysis

FFPE blocks were scanned using a micro-CT scanner (Nikon Metrology NV, Leuven, Belgium) for 8 h to obtain WBI as previously described [17]. Image slices were reconstructed using modified Feldkamp-filtered back-projection algorithms with CDPro3D (Nikon Metrology). The reconstructed imaging data were visualized and analyzed using VG Studio M2.2.6 (Volume Graphics GmbH, Heidelberg, Germany) and Dragonfly 4.2 (ORS, Montreal, QB, Canada) (the representative reconstructed images are shown in Supplementary Video S1). WBIs were subsequently digitally colorized to simulate H&E images. The same pathological findings were evaluated using WBI following the WSI review. When additional review using WBI was needed, reviewers requested recut slides of the corresponding FFPE blocks. The diagnoses of TD and PNI were made in the same manner for the WSI review. The diagnosis of LVI was made when reviewers confirmed tumor invasion with histological lymphovascular structures and their branches. CRM was measured on the same plane as the corresponding WSI and the shortest distance in the 3D direction. A lymph node (LN) was considered when reviewers recognized the LN capsule, cortex, medulla, and germinal center. An MLN was diagnosed when structural irregularities, space-occupying lesions, and density differences were identified.

### 2.4. Statistical Analysis

Categorical data were expressed as the frequency with percentage and analyzed using the χ^2^ test or the Fisher’s exact test, whereas continuous data were expressed as the mean with standard deviation (SD) and analyzed using the paired *t*-test. Tumor staging using WSI and WBI was evaluated on a case-by-case basis. The total number of BLNs and MLNs was counted in all reviewed H&E slides for WSI and FFPE blocks for WBI. Statistical significance was defined as a *p*-value < 0.05 with two-tailed tests. Statistical analysis was performed using EZR (version 1.53; Saitama Medical Center, Jichi Medical University, Saitama, Japan) [26].

## 3. Results

### 3.1. Patient Characteristics

The patient characteristics are summarized in Table 1. The pretreatment histology of included cases was adenocarcinoma (two cases of well-differentiated, eight moderately differentiated, one poorly differentiated, and two mucinous adenocarcinoma). Among them, 69.2% of cases were stage T3, and LN metastasis was seen in 46.2% of cases. Neoadjuvant chemoradiotherapy was administered to eight patients (61.5%) with a mean radiation dose of 48.4 Gy. Low anterior resections and abdominoperineal resections were performed in eight and five patients, respectively. Detailed information about included cases is listed in Supplementary Table S1.

### 3.2. Pathological Tumor Staging

**Figure 1:**
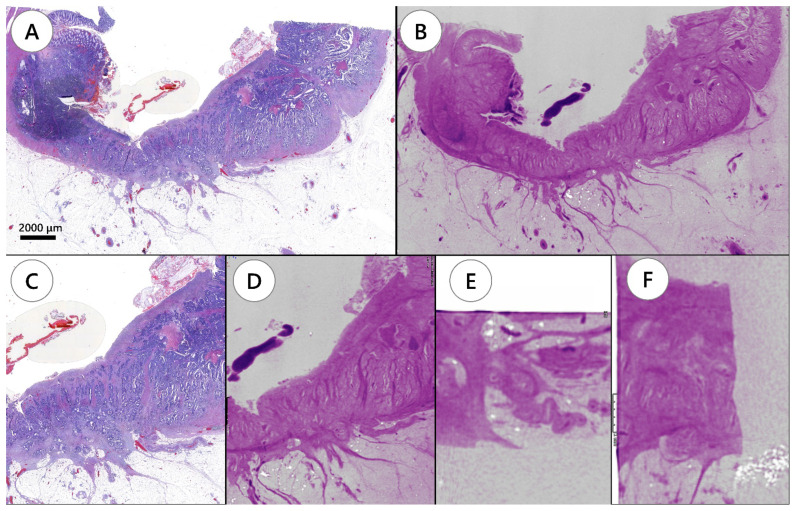
Tumor depth evaluation by conventional hematoxylin and eosin slide image and whole-block imaging. (**A**) Hematoxylin and eosin slide showing a tumor invading the subserosa. (**B**) Whole-block imaging showing subserosa invasion on the same plane of the corresponding hematoxylin and eosin slide. (**C**) Magnifying image of invaded area. (**D**) The corresponding image of whole-block imaging. Serial images are applicable for whole-block imaging through the formalin-fixed paraffin-embedded block. (**E**,**F**) Whole-block imaging showing serial images in three dimensional directions.

WSI showed the tumor site in 110 of the 237 H&E slides. In tumor staging using WSI, one case was Tis, one case was T1, two cases were T2, and seven cases were T3. The remaining two cases were diagnosed as complete response after neoadjuvant chemoradiotherapy. WBI showed similar images to WSI (Figure 1) and identified all tumor sites that were detected using WSI, resulting in the same tumor staging. Although it did not change tumor staging of the case, WBI detected an additional site of submucosal tumor invasion in one of the FFPE blocks, which was not visible in WSI.

### 3.3. Circumferential Resection Margin

After excluding two cases of complete response, the average CRM measured on the basis of WSI was 7.7 mm. In contrast, WBI showed 7.5 mm on the same plane as the corresponding FFPE block, which was not significantly different between WSI and WBI (*p* = 0.07). However, the shortest distance in the FFPE block detected in the 3D direction using WBI was 6.6 mm, which was significantly closer to the soft-tissue edge (*p* < 0.01).

### 3.4. Tumor Deposits, Lymphovascular Invasion, and Perineural Invasion

TD was detected using WSI in three cases with 17 H&E slides, and WBI showed TD in the same three cases (Table 2).

**Figure 2:**
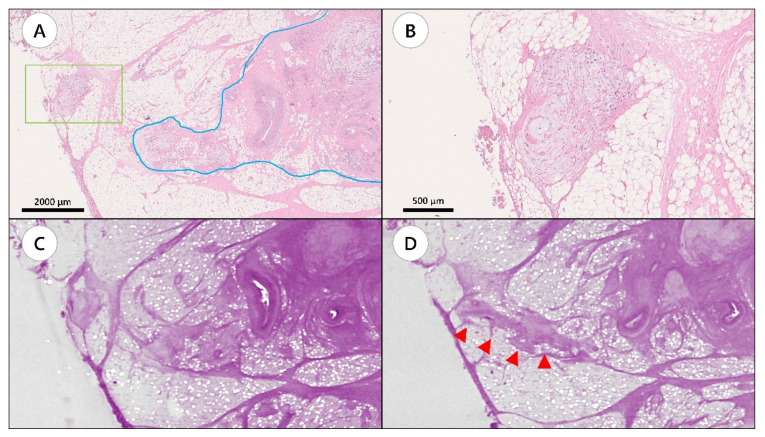
A representative case showing tumor connection between tumor deposit and primary tumor site by whole-block imaging. (**A**) Hematoxylin and eosin (H&E) slide showing tumor deposit (green box) and the primary tumor site (blue line). (**B**) Magnified image of the area in the green box. (**C**) Whole-block imaging of slide A. (**D**) Serial images of the block. Tumor connection was observed (arrow heads).

However, tumor interaction with the surrounding tissues, such as primary tumor, MLN, LVI, or PNI, was revealed using WBI, thus canceling the findings of TD in 13 H&E slides represented by 13 FFPE blocks (Figure 2, Supplementary Video S2).

LVI was detected using WSI in seven cases represented by 107 LVIs in 55 H&E slides. LVI was detected using WBI in seven cases represented by 96 LVIs in 53 FFPE blocks. WSI tended to detect more LVI findings that did WBI. However, there was no significant difference between the two methods on a case-by-case basis (seven vs. seven, *p* = 1.00).

A total of 36 PNI findings in four cases were observed using WSI, while 26 PNI findings in two cases were detected using WBI. WBI tended to detect fewer PNI findings compared to WSI. However, there was no significant difference between the two methods on a case-by-case basis (four vs. two, *p* = 0.35).

### 3.5. Lymph Node Evaluation

**Figure 3:**
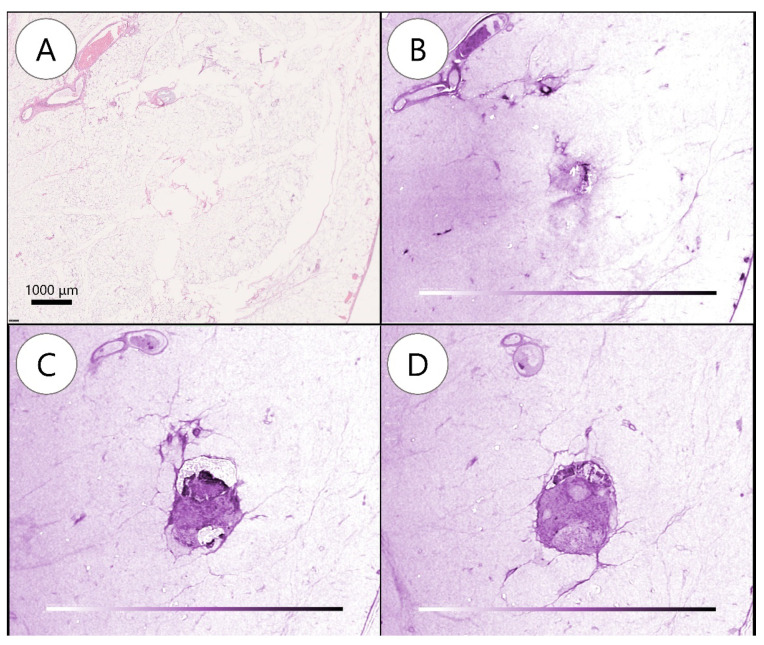
Metastatic lymph node detected by whole-block imaging. (**A**) Metastatic lymph node (MLN) not noted on hematoxylin and eosin slide. (**B**) MLN not noted on whole-block image. (**C**) Serial images showing subcapsular transparency and clusters of cells. (**D**) Structural irregularities, space-occupying lesions, and density differences deeper in (**C**).

A total of 109 LNs were detected using WSI, while 143 were detected using WBI. Eventually, WSI and WBI showed 80 and 108 BLNs and 29 and 35 MLNs, respectively. Furthermore, one LN was regarded as no LN in WSI, while malignant findings were detected in WBI (Figure 3, Supplementary Video S3). There was no significant difference between the two methods on a case-by-case basis (four vs. four, *p* = 1.00).

## 4. Discussion

To the best of our knowledge, this is the first study to show the efficacy of WBI in rectal cancer. We demonstrated that WBI of rectal resection whole-mount specimens provided additional information compared to that obtained using WSI only. WBI changed the pathological diagnosis of TD in WSI by finding surrounding tissue interactions. Furthermore, WBI showed shorter CRM and detected more BLNs and MLNs, which means that WBI could affect decision making for adjuvant treatment after surgery in such patients. These results suggest that WBI could support accurate pathological evaluation of patients with locally advanced rectal cancer.

The extent of a tumor is a significant prognostic factor in colorectal cancer [27]. Neoadjuvant chemoradiotherapy for locally advanced rectal cancer is considered one of the standard treatments [13]. These cases often show fragments of residual disease or even eradicate the tumor in their resection specimens, in which thorough sectioning and careful examination to determine the correct tumor stage are required [28,29,30]. According to the results of our study, WBI successfully identified all tumor sites that were detected by WSI. Furthermore, WBI enabled evaluation of the distribution of tumors within the FFPE blocks, revealed the relationship of the tumors with surrounding LVI, PNI, or MLN, and eventually resulted in the change of the pathological diagnosis of TD which was diagnosed using WSI. Our study showed a 76.5% decrease in TD diagnosis after referring to 3D structures of WBI. TD is considered a significant adverse prognostic factor [31]. In addition, correction of TD diagnosis can be beneficial to tumors whose distance to the surgical margin should be measured with high precision. According to the current guidelines, CRM is considered positive when the measurement is ≤1 mm and suggests an increased risk of local recurrence, distance metastasis, and poor prognosis [32]. However, there is still an ongoing search for more implications, such as tumors ≤ 0.4 mm that may have a worse prognosis [33,34]. Although the difference in CRM between WBI and WSI was approximately 1 mm, our results showed that WBI significantly improved the cutting margin assessment compared with WSI.

Presence of LVI has been reported as a high-risk factor for metastasis to LNs and other organs, and PNI has been associated with poor prognosis even in a neoadjuvant setting [35,36,37,38]. Our study showed that WBI tended to be inferior to WSI in terms of detecting LVI and PNI because of its limited resolution. In our study, WBI could identify 89.7% of LVI and 72.2% of PNI among positive cases in WSI. However, WBI could provide additional information of the connections between the primary tumor site and LVI or PNI in several cases. Although the future development of micro-CT may be expected to resolve these issues, it is important to understand that WBI provides complementary pathological information regarding LVI and PNI.

MLN is the most important prognostic factor associated with overall survival [39]. It has been shown that the chance of finding MLNs increases with the number of nodes found. Since the probability of detecting MLNs does not change after 12–15 nodes, at least 12 total LNs are required to improve metastasis detection [40]. However, it is important to identify and sample all regional LSs that can be found in a specimen and not stop at 12 [41]. Several factors affect this total number, such as surgery technique, patient features, and neoadjuvant therapy [42,43]. Despite pathologists’ persistent efforts, there are still reports of inadequate numbers [44,45]. WBI showed all BLNs and MLNs that were represented in the WSI. Moreover, WBI revealed 28 additional BLNs and six MLNs, which were not reported by WSI. The whole-mount specimen contains all the perirectal tissue, which means that WSI can reveal all LNs inside the FFPE blocks. Although WBI did not change the N stage diagnosis in this study, it showed a potential to change the TNM staging classification by providing information on the numbers and specifications of LNs inside the FFPE blocks without the need to recut slides.

There were some limitations in this study. First, this pilot study was retrospectively conducted in a single center with a small number of cases. Thus, prospective studies should be conducted to confirm our study results. Second, we did not perform serial sectioning until FFPE blocks were fully consumed; therefore, a thorough comparison between WBI and corresponding WSI findings was not completed. However, WBI may provide additional information inside FFPE blocks that is difficult to search by conventional pathological approach due to substantial burdens. Third, the included cases were not consecutive. Although we additionally searched for differences between cases with and without neoadjuvant therapy, no statistical difference was observed (Supplementary Table S2). However, further studies should be conducted prospectively with clear inclusion criteria.

## 5. Conclusions

WBI by micro-CT was found to be a nondestructive imaging method that could confirm conventional pathological features and provide additional information in patients with locally advanced rectal cancer. The combination of WSI and WBI could contribute to accurate pathological assessment.

## Figures and Tables

**Table 1:** Clinicopathological characteristics of patients with locally advanced rectal cancer.

Characteristic	*N* = 13
Mean patient age, years	58.8 ± 17.9
Sex, *n* (%)	
Male	9 (69.2)
Female	4 (30.8)
Pretreatment histology, *n* (%)	
Well-differentiated	2 (15.4)
Moderately differentiated	8 (61.5)
Poorly differentiated	1 (7.7)
Mucinous	2 (15.4)
Clinical tumor stage ^†^, *n* (%)	
cT2	3 (23.1)
cT3	9 (69.2)
cT4	1 (7.7)
Lymph node metastasis, yes, *n* (%)	6 (46.2)
Neoadjuvant chemoradiotherapy, yes, *n* (%)	8 (61.5)
Mean radiation dose ^‡^, Gy	48.4 ± 2.8
Resection, *n* (%)	
LAR	8 (61.5)
APR	5 (38.5)

^†^ Tumor staging was clinically made according to the American Joint Committee on Cancer TNM classification (eighth edition); ^‡^ Radiation dose was calculated in six patients who underwent neoadjuvant chemoradiotherapy. LAR, low anterior resection; APR, abdominal perineal resection.

**Table 2:** Pathological review comparison between conventional whole-slide imaging and combination of whole-slide with whole-block imaging.

	WSI	WSI + WBI	*p*-Value
Tumor deposit, yes, *n* (%)	3 (23.1)	3 (23.1)	1.00
Lymphovascular invasion, yes, *n* (%)	7 (53.8)	7 (53.8)	1.00
Perineural invasion, yes, *n* (%)	4 (30.8)	2 (15.4)	0.35
Lymph node metastasis, yes, *n* (%)	4 (30.8)	4 (30.8)	1.00
Total number of BLN ^†^	80	108	NA
Total number of MLN ^†^	29	35	NA

WSI, whole-slide imaging; WBI, whole-block imaging; BLN, benign lymph node; MLN, metastatic lymph node; NA, not assessed. ^†^ BLNs and MLNs were counted for all reviewed 237 H&E slides for WSI and 237 formalin-fixed paraffin-embedded blocks for WBI.

## Data Availability

The datasets used during the current study are available from the corresponding author on reasonable request.

## Supplementary Materials

The following supporting information can be downloaded at https://zenodo.org/record/6469086\#.Yl4x7dNBxPZ: Table S1. Details of patient background; Table S2. Pathological finding changes by a combination of whole-slide imaging and whole-block imaging between cases with and without neoadjuvant therapy; Supplementary Video S1. The representative reconstructed three-dimensional serial images; Supplementary Video S2. A representative case showing a connection between tumor deposit and primary tumor site by whole-block imaging. The left window shows the hematoxylin and eosin (H&E)-stained slide of the whole-mount specimen. The upper right window represents a magnified H&E image of the orange box. The lower right window shows serial images of the corresponding H&E image of the orange box and reveals the connection between the tumor deposit on the H&E slide and primary tumor site; Supplementary Video S3. Metastatic lymph node inside the formalin-fixed paraffin-embedded block detected by whole-block imaging. The video shows a three-dimensional view of a specific area of the block reviewed at several angles. There is a millimetric lymph node below the cutting surface that has metastatic features. A metastatic lymph node is not noted on the hematoxylin and eosin slide (upper left blue box). The serial images in the three-dimensional directions clearly show findings of metastasis. Therefore, whole-block images enable evaluation of the tumor in three-dimensions.
